# Determining the effect of frailty on survival in advanced ovarian cancer: study protocol for a prospective multicentre national cohort study (FOLERO)

**DOI:** 10.2340/1651-226X.2025.42292

**Published:** 2025-02-05

**Authors:** Daniel Hunde, Niklas Ekerstad, Mihaela Asp, Päivi Kannisto, Madelene Wedin, Charlotte Palmqvist, Pernilla Dahm-Kähler, Yvonne Brandberg, Mirna Abraham-Nordling, Kristina Åhlund, Vilhelm Mörlin, Nina Groes-Kofoed, Sahar Salehi

**Affiliations:** a Department of Women’s and Children’s Health, Division of Obstetrics and Gynecology, Karolinska Institutet, Stockholm, Sweden; b Department of Pelvic Cancer, Karolinska University Hospital, Stockholm, Sweden; c Department of Health, Medicine and Caring Sciences, Linköping University, Linköping, Sweden; d Department of Clinical Science, Division of Obstetrics and Gynecology, Lund University, Lund, Sweden; e Department of Obstetrics and Gynecology, Skåne University Hospital, Lund, Sweden; f Department of Biomedical and Clinical Sciences, Linköping University, Linköping, Sweden; g Department of Obstetrics and Gynecology, Linköping University Hospital, Linköping, Sweden; h Department of Obstetrics and Gynecology, Institute of Clinical Sciences, Sahlgrenska Academy, University of Gothenburg, Gothenburg, Sweden; i Department of Gynecology and Obstetrics Sahlgrenska University Hospital, Gothenburg, Sweden; j Department of Oncology and Pathology, Karolinska Institutet, Stockholm, Sweden; k Department of Molecular Medicine and Surgery, Karolinska Institutet, Stockholm, Sweden; l University West, Trollhättan, Sweden; m NU Hospital Group, Trollhättan-Uddevalla, Sweden

**Keywords:** Frailty, ovarian neoplasm, cytoreductive surgery, survival

## Abstract

**Background and purpose:**

There is an urgent need to improve patient-selection to surgical treatment in advanced ovarian cancer as our results showed that cytoreductive surgery was without effect or even detrimental in a yet unknown subgroup of women. With an ageing population, 30% of women with advanced ovarian cancer in Sweden are >75 years. Nevertheless, there are no recommendations on patient-selection, albeit treating an unselected population in a public and centralized health care setting. Little attention has been placed on frailty assessments in oncology, despite their potential to stratify the risk of adverse outcome and mortality. Consequently, we hypothesize that frailty is a predictor of poor survival.

**Patients and methods:**

In this Swedish multi-centre prospective cohort study, where the exposure is frailty, consecutive women with advanced ovarian cancer scheduled for surgery with curative intent are eligible for inclusion. Three different frailty instruments are evaluated preoperatively, blinded to the caregiver. The primary outcome is 2-year overall survival. With a fixed sample size of 450 patients, a two-sided α of 0.05 and β of 0.20, the study is powered to detect a difference in 2-year survival of 12.5% by frailty, assuming a 20% prevalence of frailty.

The result of the study will have a direct impact on clinical management and patient-selection as the results are expected to have a high external validity.

Total study-time is 5 years, with 3 years of accrual. All participating centres started accrual by September 2024. Presentation of data on primary outcome is expected 2029.

**Study registration:**

ClinicalTrials.gov NCT06298877

## Introduction

The hallmark of epithelial ovarian cancer is exfoliation to the peritoneal cavity with extensive seeding to the peritoneum resulting in carcinomatosis. Moreover, symptoms are vague and indistinct. Accordingly, the majority of patients are diagnosed at an advanced stage [1]. Surgery and chemotherapy combined remain the cornerstones in the available treatment armamentarium [2].

Complete macroscopic resection with no gross residual tumour at time of cytoreductive surgery confers the best chance of prolonged survival and is therefore the goal of the surgical procedure [3, 4]. In patients where the likelihood of achieving complete macroscopic resection is low and who are poor surgical candidates, the treatment strategy may include neoadjuvant chemotherapy before cytoreductive surgery followed by postoperative chemotherapy, chemotherapy alone or best supportive care. In Sweden, approximately 70% of women with advanced ovarian cancer are subjected to surgery as part of their initial treatment [5]. To achieve complete macroscopic resection, extensive surgery in all quadrants of the abdomen is often necessary, including multiple organ resections and resection of large areas of the peritoneum. In 50% of patients resection of any part of the gastro-intestinal tract is needed, where 11% receive a temporary defunctioning intestinal stoma [5, 6].

Avoiding peri- and postoperative complications that delay the administration of adjuvant chemotherapy is moreover imperative, as the theoretic rational behind surgical cytoreduction is exclusively attributed to enhancing the effect of chemotherapy [7, 8]. Nevertheless, despite a seemingly successful primary treatment in complete remission, most patients will recur within 2 years and the prognosis remains poor [9].

In Sweden, the health care system has been structured with centralized care and specialist surgeons to minimize the risk of residual disease and to improve survival in ovarian cancer. Notwithstanding these efforts, we demonstrated that when treating an unselected accounted for population within a public health care system setting, survival was not improved despite increasing the complete macroscopic resection rate significantly [5]. Naturally, patient selection to treatment differs by type of health care system. In a publicly available health care system, treating the general population, patient selection is imperative to avoid both toxic treatment without effect for the individual and also not to exhaust the health care system with ineffective treatment. Nevertheless, there are no guidelines for selection of patients to surgical treatment apart from expert opinion.

The complexity and dynamics presented by age-dependent physiological changes, comorbidities and newly diagnosed conditions combined are not easily synthesized to base clinical decisions on; however, these variables are all included in the concept of frailty.

Frailty is defined as a state of vulnerability secondary to a general decline in physiologic function in multiple organ systems and lack of resilience [10]. The concept of frailty is to include the complexity of the relationship between aging, comorbidities and medical condition to nuance the vulnerability of an individual. Simplified, the frailty of an individual may be described by her ability to withstand stressors [10]. Frailty is considered to increase with age but is not synonymous with old age and there are chronologically young individuals who are frail and vice versa [11]. Measurements of frailty were first introduced to aid diagnosis and care planning by screening and assessment in geriatrics [12]. Assessments of frailty to predict adverse outcomes in different medical disciplines including interventional or surgical are, however, rapidly gaining interest, where associations between frailty and adverse outcomes or mortality have been established [11, 13–16]. Comparatively, little attention has been focused on frailty assessment in oncology despite the potential to stratify risk and adverse outcome. There are several different frailty instruments, however, none has yet been considered a gold-standard why different studies have utilized different instruments.

In gynaecologic oncology different frailty instruments have been assessed, mostly by retrospective design and in single institution settings [17–20]. Nonetheless, with the promise of better selection of patients to surgical treatment, especially in advanced ovarian cancer, where it has been suggested that frailty is an independent predictor of postoperative complications, death and overall survival [21, 22].

Early reversal of temporary defunctioning intestinal stoma have been demonstrated to be safe in patients with colorectal cancer [23]. Accordingly, early stoma reversal may also be of benefit for patients with advanced ovarian cancer to reduce morbidity, but also to avoid interrupting consolidation adjuvant therapies to allow for stoma reversal surgery after completion of adjuvant chemotherapy.

Taken together, it is evident that patient selection to treatment in advanced ovarian cancer needs to urgently improve and that frailty assessment holds the promise to predict adverse outcomes and death. Nevertheless, there is no universally accepted frailty instrument. For this reason, we hypothesize that frailty can predict short-term survival and test this in a Swedish national prospective cohort study where both the phenotypic and cumulative deficit frailty models are represented. We furthermore aim to assess the feasibility of early reversal of intestinal stoma.

### Patients, methods and procedure

The FOLERO study (Frailty, quality Of Life and Early Reversal of temporary defunctioning stoma in Ovarian cancer) is a national prospective observational cohort study.

Patients with advanced ovarian cancer scheduled for surgery with curative intent are recruited at Sahlgrenska-, Skåne-, Linköping- and Karolinska University hospitals in Sweden. Consecutive patients will be assessed for eligibility and are included after written and oral informed consent. A research nurse with training in frailty assessment will conduct the comprehensive frailty assessments, which are blinded to the caregivers. The participating patients are then followed by review of hospital records, by crosscheck of vital status to the Swedish Quality Registry of Gynecologic Cancer (SQRGC) and by conventional mail 3 and 12 months postoperatively to complete patient reported outcomes. The inclusion criteria include: patients with advanced ovarian cancer scheduled for cytoreductive surgery with curative intent, age ≥18 years, signed written informed consent. Exclusion criteria include: not able to understand the Swedish or English language, diagnosis other than ovarian cancer on final pathology.

**Figure 1:**
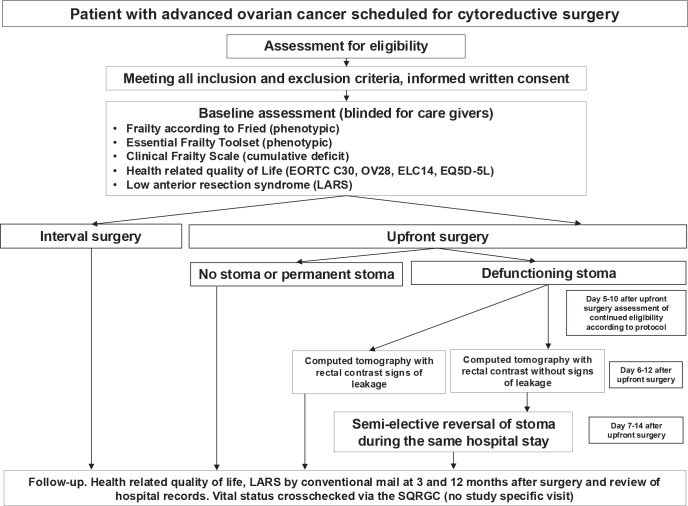
FOLERO study schema. SQRGC, Swedish Quality Registry of Gynecologic Cancer.

As the actual number of patients who receive a temporary defunctioning stoma is very low, the study comprises a small phase I interventional single arm trial conducted at the site of Karolinska only to assess the feasibility of early reversal of temporary intestinal stoma after cytoreductive surgery for ovarian cancer. In addition, participating patients also consent to the collection of tumour samples as the FOLERO study is also exploited for collection of tumour samples for translational prognostic biomarker research, not presented herein (see Figure 1 for detailed study schema).

### Primary outcomes

- 2-year overall survival, defined from the date of surgery to the date of death (any cause), or date of last follow-up for patients still alive. The 2-year cut-off was chosen based on the median survival of patients in the Stockholm/Gotland Region in Sweden who were considered ‘fit for surgery’, however in whom cytoreductive surgery was not attempted and who followed with the alternative treatment to surgery, chemotherapy only (unpublished data).
- Feasibility of early stoma reversal as defined by: (1) Anastomotic leakage rate should not exceed 6.5% [24]; (2) Urgent re-operations should not exceed 8.4% [24].

### Selected secondary outcomes

- The effect of sarcopenia on postoperative complications and survival, measured by preoperative routine computed tomography by different software programs, complications measured by the validated instrument [25].
- Description on the health-related quality of life (HRQoL) and low anterior resection syndrome (LARS) after surgical treatment assessed by validated questionnaires; European Organization for Research and Treatment of Cancer (EORTC) Core Questionnaire (QLQ-C30) + the Ovarian Cancer Module (QLQ)‐OV28 + the Elderly Module (EORTC-QLQ-ELD14), EQ‐5D‐5L, LARS questionnaire [26–30].
- The effect of frailty on the standard regimen of adjuvant chemotherapy

### Exposure

- Frailty according to the Clinical Frailty Scale-9 [31].
- Phenotypic frailty according to Fried [32].
- Phenotypic frailty according to the Essential Frailty Toolset [13].

### Control

- No frailty according to the Clinical Frailty Scale-9 [31].
- No phenotypic frailty according to Fried [32].
- No phenotypic frailty according to the Essential Frailty Toolset [13].

### Co-variables

Confounders and covariates are predefined and include known preoperative variables used presently in the overall assessment when deciding on surgical treatment and may have a known association with the outcome (survival): Age, International Federation of Obstetrics and Gynecology (FIGO) stage, Timing of surgery (upfront or after neoadjuvant chemotherapy), Eastern Cooperative Oncology Group (ECOG) performance status, preoperative plasma or serum-albumin. The co-variables will be included in the Cox regression model to adjust the estimate and are included in the monitoring plan to avoid missing data that will not be imputed. The primary outcome is cross-linked against the SQRGC/Swedish Population Register, ensuring complete coverage.

### Statistical considerations

#### Power calculation

The clinical margin for a worse short-term survival in the FOLERO study is defined as >12.5% at 2-years after surgery. To show a 12.5% reduction in 2-year survival from the expected 75% in the control group (not frail), to 62.5% in the frail group, and by assuming that the prevalence of frailty is 20% (based on previous studies), the FOLERO study needed to observe 192 events. With a total of 450 patients – recruited for 3 years and with 2 years of follow up – and with a two-sided level of significance (α) of 5%, the power (1–β) will be >80%. This corresponds to detecting a hazard ratio of 1.63.

#### Sample size

Among the four participating University hospitals at least 250 women are surgically treated annually. With a realistic estimation of a drop-out rate of 40%, it is feasible that 150 women are recruited annually over 3 years to reach the target sample size that is fixed and set to 450 women. However, the follow-up time and exact power may vary depending on the actual prevalence of frailty (see Table 1).

**Table 1:** The relationship between the prevalence of frailty and power, events and study time in the FOLERO study.

Sample size	Accrual time	Follow-up time	Total study time	Frailty prevalence %	Hazard ratio	No. of events*	Power %
**450**	3	2	5	**20**	1.63	192	83
**450**	3	1	4	**25**	1.63	149	79
**450**	3	2	5	**25**	1.63	196	87
**450**	3	1	4	**30**	1.63	153	83
**450**	3	2	5	**30**	1.63	200	90
**450**	3	1	4	**35**	1.63	157	85
**450**	3	2	5	**35**	1.63	203	92

FOLERO: Frailty, quality Of Life and Early Reversal of temporary defunctioning stoma in Ovarian Cancer.

* Represents number of deaths.

#### Interim analysis

One interim analysis will be performed 2 years after the study start or when 200 patients have been recruited to the study, to evaluate patient recruitment and the prevalence of frailty.

#### Phase I single arm feasibility trial on early stoma reversal

The statistical analysis is descriptive. In the Region of Stockholm/Gotland Sweden 11% of patients receive a temporary defunctioning intestinal stoma during cytoreductive surgery which translates to about 10 patients/year [6]. For this reason, we estimate a very small sample size of 10 patients over 3 years.

## Ethical declarations and trial registry information

The FOLERO study is managed, overseen and monitored according to Good Clinical Practice by the Clinical Trials Office/Centre for Clinical Cancer Research, Theme Cancer, Karolinska University Hospital, Sweden. Collection of data poses a potential risk of breach of personal integrity. This risk must be balanced against the knowledge produced by the research conducted. We believe that the study is of imperative importance for this patient group. To minimize the risk of breach of personal integrity, data is collected in a secure location at the university hospitals which is accessible only to health care professionals under secrecy according to the Swedish Health and Medical Care Act. All data is entered to a web-based encrypted electronic case report form system (Pheedit, version 3.03.019), pseudonymized with the code-key locked at research facilities within the University hospital accessible only to authorized personnel. Both the database server and web server are managed by Region Stockholm, Sweden IT department according to ISO standards. The study was approved by the Swedish Ethical Review Authority 2023-10-25, Dnr 2023-04696-01 and after amendment 2024-08-26, Dnr 2024-04499-02.

The study is registered at ClinicalTrials.gov NCT06298877.

## Discussion

According to Statistics Sweden, the average life expectancy of women in Sweden was 84.8 years in 2021 and is expected to increase during the coming decades [33]. With an aging population, in diagnoses with a risk of deadly outcome and where the treatment itself can be debilitating and lethal, such as cancer, age is of particular importance. When treatment prolongs survival rather than cures the fatal condition, as in advanced ovarian cancer, age is essential. However, chronological upper age limits come with risk of excluding patients in good condition from life-prolonging treatment. Accordingly, age is an unprecise variable to base clinical decisions on. It is moreover noteworthy that it is stated as part of the Swedish Health and Medical Care Act that age in itself may not decide treatment or the quality of treatment [34, 35].

With the FOLERO study we investigate generalizable clinical tools, with barely any additional cost that may directly be implemented in clinical practice to improve patient selection to surgical treatment. Accordingly, extensive surgical treatment with its associated risks may be omitted in favour of chemotherapy only in patients with very poor prognosis. The secondary outcomes generate possible hypotheses that may further improve patient selection to treatment. Furthermore, we map, for the first time in a nationwide population, the HRQoL and self-reported bowel symptoms in patients with advanced ovarian cancer who undergo surgery, which brings a chance to identify our patients’ needs and thereby improve healthcare. Finally, the study contributes by defining the feasibility of early reversal of temporary defunctioning intestinal stoma after cytoreductive surgery for ovarian cancer.

## Status of the study

In September 2024 the FOLERO study was active and recruiting in all participating University Hospitals in Sweden. To date 57/450 patients have been included.

## Data Availability

As this is a study-protocol and data has yet to be collected, assembled and analysed, data sharing is not applicable. Results from the FOLERO study will be published, data on an aggregated level will then be made available upon reasonable request.
